# Effect of an educational program about mentorship competencies on nurse mentors’ performance: a quasi-experimental study

**DOI:** 10.1186/s12912-023-01597-y

**Published:** 2023-11-14

**Authors:** Heba Moussa Hagrass, Sanaa Abd El-Azeem Ibrahim, Rasha Ibrahim El-Sayed Anany, Heba E. El-Gazar

**Affiliations:** Faculty of Nursing, Port Said University, Port Said, Egypt

**Keywords:** Nursing education, Mentorship, Mentors, Nursing students, Competencies, Performance

## Abstract

**Background:**

Mentorship is a vital part of the nursing profession to improve role transition, job satisfaction, and job retention while facilitating socialization, emotional well-being, and the acquisition of new skills.

**Aim:**

The present study aimed to evaluate the effect of an educational program about mentorship competencies on nurse mentors’ performance at Port Said Healthcare Authority hospitals.

**Methods:**

A quasi-experimental study design (pre-test and post-test one group) was used to conduct the study at seven Healthcare Authority hospitals in Port Said Governorate, Egypt. The study subjects were consisted of a purposive sample of 30 nurse mentors and 60 intern nursing students. Data were collected using three tools consisted of the Mentor Knowledge Questionnaire, Mentor Competencies Instrument (MCI), and Nurse Mentor Performance Assessment. Data analysis was performed using SPSS version 20, Student’s t-test was used to measure differences between the pretest and post-test, and Qualitative categorical variables were compared using the chi-square test. A significant level value was considered when the p-value ≤ 0.05, and Cohen’s d was used to measure the effect size.

**Results:**

the post-test scores of mentorship competencies and performance after implementation of the educational program significantly improved in the studied nurse mentors 56.1 ± 13.2, 60.5 ± 4.9 compared with pre-test scores with 37.1 ± 4.1, 49.7 ± 6.9 (P < 0.001). As determined by Cohen’s d test, the effect size of an educational program is quite large on the nurse mentors’ performance.

**Conclusion:**

The educational program about mentorship competencies was significantly improve mentorship performance of the studied nurse mentors. The study recommended dissemination and generalization of the new and innovative mentorship program to the different stages of nursing education to foster the continued growth and development of nurse mentors and nurse students. Also, recommended developing a valid mentor assessment instrument consisting of and specific to the Egyptian context to assess the Egyptian mentorship model.

**Trial Registration Number (TRN):**

The study protocol was approved by the Research Ethics Committee of the Faculty of Nursing, Port Said University (code number: NUR 13/2/2022) [10].

## Background

Practical training makes up a sizeable portion of the nursing degree education and offers students the chance to develop essential practical skills [2]. Nursing students complete this component of their development in actual clinical settings under the supervision of licensed nurses [3]. The nursing profession needs mentorship to increase role transition, job satisfaction, and job retention while promoting sociability, emotional well-being, and the acquisition of new skills [4].

According to operational definitions, mentoring is a relationship in which one person imparts their expertise and experience to the other. Research has shown that mentoring has a favorable impact on both academic performance and career development [5]. Zainol and Salam [6] described the establishment of a mutually beneficial connection between a mentor and mentee, together with the formulation of goals that are intended to benefit the mentees, as the fundamental elements of a mentoring program. Rogers, et al. [7]; Perumal, and Singh [8] asserted that the benefits of the mentorship relationship are: increased scholarly confidence, career advancement and expanded thinking, skills development, goal setting, and action planning.

In a specific educational unit, nurse mentors who work with nursing students do not always feel supported. Additionally, mentors might not have the educational background necessary to teach nursing students, which includes mentoring, ensuring competency with necessary activities and abilities, giving prompt feedback, setting goals, and developing critical thinking [9]. Understanding the role and responsibilities of a mentor which includes, clinical education teaching, student assessment, goal orientation, how to conduct a student-centered evaluation, techniques for providing feedback, and successful communication with the student are all part of mentoring education. Students believed that having regular conversations and setting goals with their mentors improved their learning capacity [10].

In their study conducted to assess the mentorship applications in nursing clinical education, Pelin et al. [1] found that mentorship systems used currently are not effective, mainly due to variations in the mentor preparation programs on offer and the mentor–student ratio in Turkey. Globally, it is obvious that mentoring systems are of utmost importance due to various problems in nursing clinical education. In their study, Foolchand [11] revealed that both students and nurses recognized that teaching, assessing, communication, managerial and leadership skills, were identified as core competencies for mentors. They also shared the same views regarding barriers to mentoring, such as staff shortage, lack of resources, and inadequate support from management.

Tuomikoski et al. [12] conducted a study on how mentoring education affects nurse mentors’ competence in mentoring students during clinical practice and concluded that the participating nurses showed after the educational intervention a statistically significant increase in their competence regarding knowledge of mentoring practices in the workplace, student-centered evaluation, identifying student needs, mentoring practices between mentor and student, supporting students’ learning processes, goal orientation in mentoring and constructive feedback. In a similar study, Ratliff [9] performed a study on an educational module to increase competence for nurse mentors who mentor nursing students on a dedicated educational unit. In their study, the intervention showed effectiveness in expanding the nurse mentors’ knowledge and increasing their competence level in certain areas, as well as the participants asserted the benefit of having mentoring education before mentoring nursing students to support the mentor and their students.

### Significance of the study

As part of their professional responsibilities, qualified and experienced nurses provide clinical mentoring to the students during their practice placement. by directing and supervising students as they carry out nursing operations, but with little focus on teaching and evaluating theoretical knowledge. Therefore, clinical student mentoring is driven by service rather than education [11]. Strengthening mentors’ competence in mentoring students and improving the quality of mentoring in the clinical learning environment are the fundamental aims of mentoring education [12].

Commonly mentors are not employed by educational bodies. Additionally, mentors are frequently not obliged to take any training in mentoring before mentoring a student [5]. Currently, there is no formal mentorship training in the health care organizations in Egypt, which may cause variability in the mentoring of nursing students. Therefore, this study aimed to implement and evaluate the effect of educational program about mentorship competencies on nurse mentors’ performance at port said universal health insurance hospitals.

### Study Aim

This study aims to evaluate the effect of an educational program about mentorship competencies on nurse mentors’ performance at Port Said Healthcare Authority hospitals. The study hypothesized that the educational program about mentorship competencies will improve the performance of nurse mentors who participate in the study.

## Methods

### Study design

A quasi-experimental study design (pretest/posttest one-group design) was utilized to achieve the aim of this study.

### Study setting

The present study was conducted at seven Healthcare Authority hospitals affiliated with Egypt Healthcare Authority – Port Said Governorate, Egypt. The study settings described as following.

**Al-Salam general hospital**, the hospital specializes in providing urgent medical care for emergency cases. The hospital capacity 85 beds, with a total of 288 nurse. **Al Nasr Children’s Specialized Hospital**, the hospital specializes in providing tertiary specialized medical services for children from the age of one day to the age of 18 years in specialized health services. The hospital also provides heart surgery and cardiac catheterization services for adults with a bed capacity of 94 beds, and a total number 312 nurses. **Al-Zohour central hospital**, the hospital specializes in providing several medical services of emergency, surgical care, and critical care with capacity of 48 beds, with total number 68 nurses.

**Obstetrics & Gynecology specialized hospital**, the hospital provides several services: “emergency for obstetrics women, obstetrics and gynecology operations, outpatient clinics. It has a capacity of 84 beds. The total number 74 of nurses. **Ophthalmic specialized hospital**, the hospital specializes in providing urgent and non-urgent surgical services, whether simple, minor, major or skilled operations of ophthalmology specialist. The hospital’s capacity is 30 beds, in addition to 7 outpatient clinics. The total number of nurses 46. **Elhayat central hospital**, the hospital specializes in providing inpatient and intensive care, whether for adults or children. The hospital capacity of 68 beds beside dialysis unit includes 27 dialysis machines, with total number nurses 273. The hospital also includes 12 different outpatient clinics. **Mogamaa Elshefaa general hospital**, the hospital specializes in providing tertiary medical services. the hospital capacity 100 beds. The total number of nurses is 320.

### Study subjects

The current study consists of two sample groups (nurse mentors and intern nursing students). The first sample group consists of nurses who were selected from the study settings to work as nurse mentors under the supervision of the Italian project for nursing support allocated in the technical health institute (branch B). They were selected through the project coordinators’ interview against settled inclusion criteria as following: the communication skills, their professional appearance (the professional image of a nurse to be a role model in this item for students), and appropriate tact of speaking were noted to set a good role model for students. The interview’s items were scored from a total 100%. The interviewed nurses’ scores were arranged in descending, and then the highest scores who were chosen.

Also, nurses who hold an administrative job (e.g., head nurses, ward managers), or on special vacations during the internship period, or work the night shift all month schedule and cannot mentor students during this shift were excluded. The total number of the selected sample group was 30 nurse mentors. The second sample group consisted of all intern nursing students who were supervised by the studied nurse mentors to assess their mentorship competencies with total number 60 intern nursing students.

### Data collection tools

#### Tool 1: this consisted of two parts as follows

***Part (I): Personal and work-related data***: The personnel and work characteristics of nurse mentors such as age, gender, level of education, department, marital status, years of experience, and previous attendance of an educational program were included in the scope of the study.

***Part (II)***: **Nurse Mentors’ Knowledge Questionnaire**: it was developed by the researcher, based on a literature review [4, 13, 14] to assess nurse mentors’ knowledge regarding nursing mentorship before and after program implementation. The questionnaire was designed in Arabic to avoid misunderstanding. It is composed of 59 multiple choice questions and covers the following domains: (1) Mentorship (the mentoring system, role and responsibilities, clinical mentor competencies, and factors affecting the learning environment), (2) Adult learning principles, (3) Mentorship skills (active listening, reflection thinking, reasoning questions), (4) Mentorship methods and tools to improve clinical understanding (learning contract, briefing & debriefing), (5) Evaluation of clinical understanding (logbook, feedback, formative mid-term evaluation, summative final evaluation).

Regarding nurse mentors’ knowledge; the answer was evaluated using a model key answer prepared by the researcher. The “correct answer” scored one, while the “incorrect answer” scored zero. For each domain, the items scores were added and divide the total score by the number of items to get the mean score for the domain. These scores were converted to percentiles and means and standard deviations were calculated. the percentages scores as cut-off points used to assess the level of knowledge. These scores were considered as satisfactory if the percent score was equal to or above 75% and unsatisfactory if less than 75% based on statistical analysis and the importance of nurses’ knowledge regarding the mentorship of nursing students [15].

##### Tool 2: Mentor Competencies Instrument (MCI)

The scale was adopted from Hasanen [13]. It consisted of 19 indicators designed to evaluate mentoring competencies by nursing students in eight factors: comprehending (2 indicators); relating (5 indicators); facilitating (2 indicators); informing (3 indicators); challenging (3 indicators); reflecting (2 indicators); motivating (1 indicator) and envisioning (1indicator). Using a Likert scale from 1 to 4, where 1 equals “poor” and 4 equals “excellent”. The item scores were summed and the total was divided by the number of items, to give a mean score for the part. These scores were converted into percent scores. Mentorship competence was high if the percent score was > 64–80%, moderate if the percent score was > 48–64%, and low if the percent score was > 32–48% [12].

##### Tool 3: Nurse mentor performance assessment

The observational checklist adapted from Foolchand [9] was used to assess nurse mentors’ performance, and consisted of 38 items under the following dimensions: (1) mentor characteristics (8 items); (2) communication (7 items); (3) goal-oriented mentoring (5 items); (4) student-centered evaluation (10 items); (5) constructive feedback (5 items); (6) Reflection (3 items). It was rated from 0 to 2. where 0 equals “not applicable”, 1 equals “not done” and 2 equals “done”. The item scores were summed and the total was divided by the number of the items and gave a mean score for the part. The performance was satisfactory if the percent score was equal to or above 75% and unsatisfactory if less than 75% based on statistical analysis and the importance of nurses’ performance regarding mentorship skills.

### Tool validity and reliability

The face and content validity of the study tools were checked by a panel of experts consisting of seven experts from the Nursing Administration Department at Mansura University, Egypt. The experts reviewed the instruments for clarity, relevance, comprehensiveness, and understanding of applicability. Comments and suggestions of the experts were considered and necessary modifications, corrections, and clarification of the items were made accordingly. The reliability of the tools used in this study was assessed by Cronbach’s alpha coefficient test to determine the internal consistency of the study tools. The internal consistency reliability for nurse mentors’ knowledge tool was 0.903, and that for mentorship competencies was 0.901. The internal consistency reliability for the nurse mentor performance tool was 0.894.

### Pilot study

Before entering the actual study, the pilot study was conducted on 10% 3 nurse mentors (from who excluded from the study for their work at the night shift schedule) and 6 intern nursing students of the sample to assess the clarity, practicability, and feasibility of the tool and to estimate the proper time required for the interview. Appropriate modifications were made according to the results of the pilot study.

### Ethical considerations

The study was approved by the Research Ethics Committee (REC), Faculty of Nursing/ Port Said University with (code number: NUR 13/2/2022) [1] based on the standard of the committee, Faculty of Nursing/ Por Said University. An official letter containing the title and the aim of the study was sent from the Dean of the Faculty of Nursing - Port Said University to the director of each setting to obtain approval from the hospital administrator for data collection in the abovementioned settings. Furthermore, written consent for participation in the study was obtained from nurse mentors and intern nursing students after clearing out all aspects of the study.

### Fieldwork

A field study was conducted for seven months from the beginning of September (2022) to the end of March (2023). The training sessions of the educational program were conducted for 9 continuous days before they supervised the internship nurse students. The studied nurse mentors apply the program during the nurse internship training in six months from October (2022) to the end of March (2023). The study was carried out through the following phases. The study was carried out through the following phases:

#### Phase I (Assessment Phase)

In this stage obtaining official permissions to carry out the study, the researcher visited the study settings and arranged with the nursing director for the actual implementation of the study. Then, nurse mentors and nurse students were recruited. The researcher clarified the sheets of the three tools to nurse mentors and nurse students and asked them to complete them. Each tool was completed in approximately 40 to 45 min.

***Phase II (Planning)***: The educational program was designed based on the assessment data collected in Phase I. The educational program aimed to enhance nurse mentors’ competencies. The handout includes theoretical content in the Arabic language to avoid misunderstanding and covers the following content: an overview of mentorship (adult learning principles, the mentoring system, roles and responsibilities, clinical mentor competencies, and factors affecting the learning environment). Mentorship skills (active listening, reflection thinking, reasoning questions). Mentorship methods and tools to improve clinical understanding (learning contract, briefing & debriefing). Evaluation of clinical understanding (logbook, feedback, formative mid-term evaluation, summative final evaluation).

***Phase III (The educational program implementation***): At the beginning, the researcher met with the whole group of the studied nurse mentors in the technical health institute (branch B) and explained the aim and procedures of the study. A copy of the handout was given to each nurse mentor to facilitate remembering the knowledge and practices during the explanation of the theoretical part. The program was presented in clear and concise form using different teaching methods while discussing the rationale and precautions for each step, such as small discussions, lectures, demonstrations, and re-demonstrations and appropriate teaching media as audiovisual material and real objects. At the end of the researcher’s demonstration, nurse mentors were asked about any unclear steps that needed repetition or explanation before the re-demonstrations. The researcher emphasized that this session was done for teaching purposes not for evaluation, so mistakes and forgetting were allowed and were corrected immediately by the researcher.

The program was implemented for nurse mentors in terms of 5 h per day for 5 days to train on the theoretical part of the program, the trainee took a 30-minute break, followed by 4 days of training in the simulation labs in the institute for training on the practical procedures included in the internship students’ logbook, in which the studied nurse mentors supervised their application by the nurse students in the hospitals of the study settings during the 6-month internship period.

#### *Phase IV* (*evaluation phase*)

The program outcome was evaluated by using the first tool for assessing the participants’ knowledge immediately after program implementation before conducting the internship training period. The 2nd tool was completed by the studied intern nursing students to assess the studied nurse mentors’ competencies, and the researcher used the 3rd tool to assess their performance during the clinical training of the internship students. The evaluation phase was performed during the last month of the internship period (the 6th month).

### Statistical analysis

The collected data were organized, tabulated, and statistically analyzed using SPSS for Windows version 20.0 (SPSS, Chicago, IL). Continuous data were normally distributed and are expressed as the mean ± standard deviation (SD). Categorical data are expressed numbers and percentages. The chi-square test (or Fisher’s exact test when applicable) was used for comparison of variables with categorical data. The reliability (internal consistency) test for the questionnaires used in the study was calculated by Cronbach’s alpha coefficient test. A significant level value was considered when the p-value ≤ 0.05. Student’s t-test was used to measure differences between the pretest and post-test. Cohen’s d used to measure the effect size was considered small effect size at < 0.5, medium effect size at 0.5 < 0.8, and large effect size at > 0.8.

## Results

### Personal characteristics of the study subjects

Table 1 illustrates that the highest percentage of the studied nurse mentors (50%) were in the age group of 24–29 years old, 83.3% of them were females, 53.3% of them were married, 56.7% of them had a Bachelor of Nursing, 33.3% of them had 5–10 years of experience, and none of the studied mentors had previous training courses about mentorship before the current study. The results indicate that the highest percentage of the studied intern nursing students (83.3%) were in the age group of 20–22 years old, and 93.3% of them lived in urban areas.

### Nurse mentors’ knowledge regarding mentorship

Table 2 shows that there was a highly significant improvement post-program in the studied nurse mentors’ knowledge compared with pre-program results in all scale domains (p < 0.001). As determined by Cohen’s d test, the effect size of an educational program is quite large on the nurse mentors’ knowledge (Cohen’s d = 5.376142). Additionally, there were statistically significant differences between the studied nurse mentors’ total knowledge pre- and post-educational program implementation (t-test 20.733).

### Nurse mentors’ competencies

As perceived by the studied intern nursing students, Table 3 clarifies that the post-program mean score for mentorship competencies revealed a significant (p < 0.001, Cohen’s d = 1.943995) increase for the studied nurse mentors’ competencies (56.1 ± 13.2) as compared with pre-program mean scores (37.1 ± 4.1). However, there was a significant difference between pre-program and post-program total mean scores (t-test = 7.530).

### Nurse mentors’ performance

As shown in Table 4, post-program mean scores clarify there were significant differences regarding the studied nurse mentors’ performance in all dimensions except communication skills (p = 0.071). However, there was an increase in the post-program total mean score (60.5 ± 4.9) as compared with the pre-program total mean score (49.7 ± 6.9) with a large effect (Cohen’s d = 2.105564). Additionally, there was a significant difference between pre and post-program mean scores (p = 0.009, t-test = 7.002).

### Relation between study variables

Table 5 elaborates that there was a statistically significant relation between the studied nurse mentors’ knowledge and their performance post-program (P = 0.008) and with their competencies post-program (P < 0.001).

**Table 1:** Personal characteristics of the study subjects

Nurse Mentors (n = 30)	n	%
**Age (years)**		
25 ≤ 29	15	50
30 < 39	11	36.7
≥ 40	4	13.3
**Gender**		
Male	5	16.7
Female	25	83.3
**Marital Status**		
Single	14	46.7
Married	16	53.3
**Educational Level**		
Technical diploma of nursing schools	6	20
Health Technical Institute Diploma	7	23.3
Bachelor of Nursing	17	56.7
**Experience (Years)**		
< 5	9	30
5 < 10	10	33.3
10 < 15	4	13.3
> 15	7	23.3
**Attending training courses on mentoring nursing students**		
yes	0	0
no	30	100
**Intern nursing students (n = 60)**	**n**	**%**
**Age (years)**		
17–19	17–19	17–19
20–22	20–22	20–22
**Residence**		
Urban	Urban	Urban
Rural	Rural	Rural

**Table 2:** Nurse mentors’ knowledge pre-, and post-program (n = 30)

Domains	Pre-program		Post-program		^*^Cohen’s d	^**^t-test	^***^ *p*
	Mean ± SD	Min-Max	Mean ± SD	Min-Max			
1. Mentorship process	6.1 ± 1.8	3–8	15.4 ± 1.2	13–17	6.0796	23.982	< 0.001**
2. Adult learning principles	1.5 ± 0.6	0–2	3.3 ± 0.9	2–4	2.353394	8.992	< 0.001***
3. Mentorship skills	4.4 ± 2.0	1–7	7.9 ± 1.1	6–9	2.168524	8.292	< 0.001***
4. Mentorship methods and tools	10.2 ± 3.1	4–16	17.9 ± 1.3	15–20	3.239414	12.570	< 0.001***
5. Evaluation of clinical understanding	2.7 ± 1.3	0–5	7.1 ± 1.2	5–8	3.517187	13.731	< 0.001***
**Total Mentors’ knowledge**	**25.0 ± 6.2**	**3–8**	**51.7 ± 3.3**	**47–57**	**5.376142**	**20.733**	< 0.001***

*Cohen’s d: small effect size at < 0.5, medium effect size at 0.5 < 0.8, large effect size at > 0.8

**t-test: student t-test comparing between pre- and post-program means ***Statistically significant at p < 0.05

**Table 3:** Nurse mentors’ competencies as perceived by intern nursing students pre-, and post-program (n = 60)

Mentorship competencies	Pre-program		Post-program		^*^Cohen’s d	^**^t-test	^***^ *p*
	Mean ± SD	Min-Max	Mean ± SD	Min-Max			
1. The skill of comprehending and listening actively	3.9 ± 0.8	3–5	6.0 ± 1.6	3–8	1.660196	6.308	0.002*
2. The skill of building relationships	8.5 ± 1.2	6–11	14.5 ± 3.5	8–19	2.293319	8.816	0.017*
3. The skill of facilitating and coaching	4.0 ± 1.3	2–7	5.8 ± 1.9	2–8	1.105731	4.319	< 0.001**
4. The skills of providing feedback and guiding	6.5 ± 1.3	5–9	9.0 ± 2.4	5–12	1.295319	5.126	0.002*
5. The skill of conflict management	5.8 ± 1.5	3–9	8.5 ± 3.0	3–12	1.13842	4.405	< 0.001**
6. The skill of reflecting	4.2 ± 1.3	2–7	5.7 ± 2.0	2–8	0.889304	3.565	< 0.001**
7. The skill of encouraging	2.4 ± 0.7	1–4	3.2 ± 0.9	2–4	0.992278	4.000	0.025*
8. The skill of solution-finding	1.8 ± 0.6	1–3	3.3 ± 0.9	1–4	1.961161	7.164	< 0.001**
**Total Mentors’ competencies**	**37.1 ± 4.1**	**31–47**	**56.1 ± 13.2**	**34–75**	**1.943995**	**7.530**	< 0.001**

*Cohen’s d: small effect size at < 0.5, medium effect size at 0.5 < 0.8, large effect size at > 0.8

**t-test: student t-test comparing between pre- and post-program means ***Statistically significant at p < 0.05

**Table 4:** Nurse mentors’ performance pre-, and post-program (n = 30)

Dimensions	Pre-program		Post-program		^*^Cohen’s d	^**^t-test	^***^ *p*
	Mean ± SD	Min-Max	Mean ± SD	Min-Max			
1. Mentor’s characteristics	9.4 ± 1.2	8–11	12.6 ± 2.0	10–16	1.940285	7.382	< 0.001**
2. Communication	9.5 ± 1.9	6–12	11.1 ± 1.9	8–14	0.842105	3.156	0.071
3. Goal-oriented mentoring	6.8 ± 1.6	5–9	8.4 ± 1.5	6–10	1.031721	3.156	0.004^*^
4. Student-centered learning	13.6 ± 2.3	10–17	16.0 ± 2.4	10–19	1.021046	3.933	0.020^*^
5. Constructive feedback	6.7 ± 1.4	3–8	8.0 ± 1.3	6–10	0.962303	3.797	0.010^*^
6. Reflection	3.6 ± 1.6	1–6	4.5 ± 1.5	1–6	0.580343	2.074	0.004^*^
**Total Performance Level**	**49.7 ± 6.9**	**36–60**	**60.5 ± 4.9**	**48–68**	**2.105564**	**7.002**	0.009*

*Cohen’s d: small effect size at < 0.5, medium effect size at 0.5 < 0.8, large effect size at > 0.8

**t-test: student t-test comparing between pre- and post-program means ***Statistically significant at p < 0.05

**Table 5:** Relation between nurse mentors’ knowledge and their performance and competencies (n = 30)

	Mentors’ knowledge							
	Pre-program				Post-program			
	Unsatisfactory Knowledge (n = 19)		Satisfactory Knowledge (n = 11)		Unsatisfactory Knowledge (n = 6)		Satisfactory Knowledge (n = 24)	
	**n**	**%**	**n**	**%**	**n**	**%**	**n**	**%**
**Mentors’ performance**								
Unsatisfactory performance	15	78.9	6	54.5	5	83.3	6	25.0
Satisfactory performance	4	21.1	5	45.5	1	16.7	18	75.0
**Test of significance (χ**^**2**^, **P)**	**χ**^**2**^ **= 1.975, P = 0.160**				**χ**^**2**^ = 7.033, P = 0.008*			
**Mentors’ Competencies Level**								
Low level of mentorship competencies	12	63.2	7	63.6	4	66.7	1	4.2
Moderate level of mentorship competencies	4	21.1	3	27.3	2	33.3	6	25.0
High level of mentorship competencies	3	15.8	1	9.1	0	0.0	17	70.8
**Test of significance (χ**^**2**^, **P)**	**χ**^**2**^ **= 0.350, P = 0.839**				**χ**^**2**^ = 15.625, P < 0.001**			

χ2: Chi square * p < 0.05 **p < 0.001

## Discussion

The current study aimed to evaluate the effect of an educational program about mentorship competencies on nurse mentors’ performance at Port Said Healthcare Authority Hospitals. The findings illustrated that there was a highly statistically significant differences between preprogram and post-program knowledge mean scores. This shows that nurses need well-designed training programs on mentorship competencies before conducting their supervision on the nurse students training in the clinical settings. If the nurse mentors’ educational needs are met, they can contribute to the improvement of nursing clinical learning and decrease the gap between theory and practice.

This result is supported by Calunsag [16], who conducted a study entitled An Education Program for Improving the Knowledge of Experienced and Aspiring Mentors in the Philippines among 16 nurse mentors and found that the knowledge post-test scores were improved after implementation of the educational program. Additionally, the study agreed with Tuomikoski et al. [12] in Finland, who studied how mentoring education affects nurse mentors’ competence in mentoring students during clinical practice between 150 nurse mentors, and reported that the participating mentors showed a statistically significant increase in their knowledge regarding mentoring practices in the workplace after the educational intervention. As well as, Oikarainen et al. [17] concluded that nurse mentors were statistically significantly more satisfied with mentoring education, and reported that it had statistically significantly higher impact on their ability and willingness to mentor students. According to Rustiawan et al. [18] nurse mentors need knowledge and guidance to develop their role according their character and abilities.

**Regarding nurse mentors’ mentorship competencies**, the present study findings revealed that there was significant improvement in the studied nurse mentors’ competencies post-program as perceived by their nurse students. The highest percentage was regarding skills of providing feedback and guidance, conflict management, and reflection. During program implementation, nurse mentors presented their interest in their new role and their feeling of its importance in nursing education, which reflected positively on their competencies during the training of nursing students and their presence as role models for them. This, in turn, was reflected in the students’ opinion of improving the competencies of their mentors. As mentioned by Ekong and Carolyne [19] Mentored student nurses experience reduced anxiety, and mentoring provides a supportive learning environment and increased self-awareness of one’s own values and beliefs, confidence, maturity and responsibility. Lack of time, dual responsibility, heavy workload, personality, and attitude may negatively impact the mentoring process. An effective mentoring program requires a relevant mentoring model and clear definitions of its context, structure, and goals.

The present result is in agreement with the study by Tuomikoski et al. [12], who found that after completion of mentoring education, competence increased significantly (p < 0.05) in all areas (Student-centered evaluation, orientation in mentoring, supporting students learning, constructive feedback, mentoring characteristics, variables of motivation and reflection). In addition, the education intervention significantly improved nurse mentors’ competence in mentoring nursing students. Also, Pramila-Savukoski et al. [20] indicated that there is scope for improving mentoring by improving organizational mentoring practices in workplaces, and increasing mentoring knowledge and resources. According to Jack et al. [21]; Lavoie‐Tremblay et al. [22] to improve mentoring in clinical learning environments, regular mentor education needs to be planned together with the goals for nurses’ individual career growth. Education helps mentors not only to develop pedagogical competences, but also to improve their knowledge of nursing curricula and bridge the theory-practice gap, which often hinders clinical learning and contributes to the shock of transition from undergraduate education to a professional nursing career.

In contrast, Ratliff [9] found that there were significant differences between pre-test and post-test scores noted in the following competency areas: guiding and assisting students in setting and obtaining clinical goals, reflection practices during mentoring, asking the student to reflect critically and holistically, and providing constructive feedback. Additionally, Mikkonen [10] studied the development and testing of an evidence-based model of mentoring nursing students in clinical practice among 1360 nurse mentors working in primary care and specialty care and confirmed that mentor education significantly improved mentors’ competencies and improved students’ clinical learning (p < 0.01).

**Regarding nurse mentors’ performance**, the study results clarified that the nurse mentor’s performance was improved post-program. The highest percentages were related to student-centered learning and constructive feedback. However, the lowest percentages were regarding mentors’ characteristics. This result highlights the importance of conducting the educational program to provides them with mentorship skills and to improve their knowledge about reflection during practice, which represents a continuous learning process for them. Additionally, the content of the educational program units meets their needs and their desire to carry out the tasks of helping students acquire nursing skills with high efficiency.

This result is consistent with Foolchand [11] who conducted a study entitled “A clinical monitoring framework for student nurses” in Mauritius. Among 255 nurses, their mentoring skills were evaluated by 115 students after the implementation of the training framework, and they found that there was a significant improvement in mentors’ performance for those skills, especially regarding communication, feedback, and student-centered learning. In addition, Russell et al. [23] and Mårtenson et al. [24] identified participation in mentoring education to positively affect participant views of clinical mentoring and working with students. Whereas, Hookmani et al. [25] emphasizes the importance of mentors training should be provided to enable them to learn new skills that align with their mentorship roles i.e., being objective, concise, skill-driven, and being able to utilize systemic analyses to overcome any ongoing nursing practice challenges with their mentee team.

However, there were highly statistically significant differences between pre- and post-program scores in all domains except communication skills, and there was a convergence between the pre- and post-program scores. The possible explanation is that the studied nurses had received on-the-job training courses about communication skills as they mentioned, which improved their communication skills with students equally pre- and post-program. A similar finding was reported by Keinänen et al. [26], who studied “Effectiveness of mentoring education on health care professionals´ mentoring competence” and reported statistically significant improvements (p < 0.01) in various aspects of mentoring skills performance namely (student-centered evaluation, identifying students’ learning needs, goal orientation in mentoring, and constructive feedback) after the mentoring education. In addition, Desai et al. [27] studied 28 mentors and concluded that there was significant improvement in mentorship skills in all domains.

The current study results showed that there was a statistically significant relation between the studied nurse mentors’ knowledge and their performance, and competencies. Additionally, there was a statistically significant relation between the studied nurse mentors’ performance and their competencies. This relation indicates the comprehensiveness and effectiveness of the educational program on nurse mentors’ competencies and performance. The present results are in agreement with a study by Spiva et al. [28], who studied the effectiveness of an evidence-based practice nurse mentor training program among 66 nurse mentors working at five hospitals located in the southeastern US, and revealed that nurse mentors’ knowledge, attitude, and skill level were improved after implementation of the training program.

Tuomikoski et al. [12] reported that participation in mentoring education was positively related to mentors’ mentoring competence and mentoring practices in the workplace with a strong impact on their motivation to practice mentoring in their daily nursing work. As well as Abdel-Samea et al. [29] found that there was positive correlation between the studied nurses’ perception and their role performance, and asserted that increasing nurses’ knowledge about their role during clinical training of nursing students could improve their performance. The present result is contrary to the study by Mikkonen et al. [10] who studied “Mentors’ competence in mentoring nursing students in clinical practice” in Italy, and proved that having completed mentoring-specific education did not significantly affect nurses’ competence in any of the seven areas of mentoring competence.

## Conclusion

The findings of the current study concluded that the educational program about mentorship competencies was effective in improving the studied nurse mentors’ performance. Through the evaluation of nurse mentors, it was found that the participants experienced satisfactory level of knowledge about the mentorship process, such as mentorship skills, methods, tools, and clinical understanding, as well as the acquisition of new information about adult learning principles. The acquisition of this knowledge was affected positively on the participants’ competencies and performance. The study recommended dissemination and generalization of the new and innovative mentorship program to the different stages of nursing education to foster the continued growth and development of nurse mentors and nurse students. Also, recommended developing a valid mentor assessment instrument consisting of and specific to the Egyptian context to assess the Egyptian mentorship model.

### Implications for practice and future directions

The findings gained from the current study can be valuable for dissemination and generalization of the new and innovative mentorship program to the different stages of nursing education to foster the continued growth and development of nurse mentors and nurse students. Moreover, the findings of this study will be beneficial for nurses, nursing educators, and clinical setting managers to design effective training programs according to the identified needs of nurse students to facilitate the transition to a realistic clinical environment. This can lead to positive social change by developing future generations, in addition to providing improved care to patients, the institution, and surrounding community. The results of the study also demonstrated that training the nurse mentors to apply the procedures in the students’ logbook can reduce the gap between theory and practice in nursing education and link clinical settings with nursing educational bodies. In addition, there is a need to develop a valid mentor assessment instrument consisting of and specific to the Egyptian context to assess the Egyptian mentorship model.

### Limitations of the study

There are certain limitations to this study. First, this study only included immediate post-test follow-up because it was applied only to nursing internship students, which takes only 6 months. Therefore, this study needs to be applied to students in other educational levels over a longer period of study in order to follow up evaluation of the results of the study. Second, all of participant students were females, which may have influenced the sample’s representativeness and the generalizability of the findings. Third, it was not possible to use randomization, participant blinding, or a control group in this study, and this issue affects the internal validity of the study. To address this concern, several procedural remedies were applied by improving the scale items’ clarity by pilot testing with nurses who had the same inclusion criteria but had already been excluded from the program due to their commitment to the night shift. Also, the inclusion of nursing students as a second study group and the researchers collected nursing students’ perceptions regarding their mentors’ competencies pre- and post-program with the second study tool as a way to overcome the concern of bias [30]. finally, as mentioned in the study results all the studied nurse mentors had no previous training regarding mentorship which excludes the effect of history factor on the study results.

## Data Availability

Due to confidentiality concerns, the data and materials used in the current study cannot be made publicly available. However, they are available from the corresponding author upon reasonable request.
